# No conclusive link between indoor fuel use and atopic dermatitis: a systematic review and meta-analysis with rigorous publication bias correction

**DOI:** 10.3389/fpubh.2025.1672618

**Published:** 2025-10-07

**Authors:** Jing Lang, Jianxun Ren, Yaqin Li, Huanhuan Qu, Gaoyong Dong, Yaning Li, Jinhe Wang, Na Lang

**Affiliations:** Department of Dermatology, Xiyuan Hospital of China Academy of Chinese Medical Sciences, Beijing, China

**Keywords:** indoor fuel, air pollution, atopic dermatitis, allergy, meta-analysis, systematic review

## Abstract

**Background:**

Household air pollution (HAP), primarily from the combustion of indoor fuels for cooking and heating, represents a major global public health challenge. Concurrently, the prevalence of atopic dermatitis is rising worldwide. However, epidemiological studies examining the association between indoor fuel use and the risk of AD have produced inconsistent findings. This study aimed to systematically evaluate the existing evidence and rigorously assess the potential impact of publication bias on this association.

**Methods:**

A systematic search of PubMed, Embase, Web of Science, and the Cochrane Library was conducted to identify observational studies assessing the association between indoor fuel use and AD risk. Odds ratios (ORs) and their 95% confidence intervals (CIs) were pooled using a random-effects model. Heterogeneity was evaluated using the I2 statistic and Cochran’s Q test. Publication bias was comprehensively assessed using funnel plot visualization and multiple statistical methods, including Egger’s, Begg’s, Peters’, Deeks’, Macaskill’s, and Tang’s tests. Sensitivity analyses and bias-correction procedures, including the trim-and-fill method and parametric selection models, were performed. Subgroup analyses were conducted to explore potential sources of heterogeneity.

**Results:**

This analysis included 10 studies, comprising 21 independent effect sizes. The initial pooled analysis revealed a weak but statistically significant positive association between indoor fuel use and AD risk (pooled OR = 1.158, 95% CI [1.051, 1.276], *p* = 0.0031), with moderate between-study heterogeneity (I^2^ = 43.0%, *p* = 0.0195). However, multiple statistical tests indicated a significant risk of publication bias (e.g., Egger’s test, *p* = 0.0006). The trim-and-fill method yielded an adjusted OR of 1.040 (95% CI [0.930, 1.164]), and parametric selection models produced similarly null results (e.g., standard selection model adjusted OR = 1.000, 95% CI [0.992, 1.008]). Subgroup analyses suggested the association was primarily driven by studies on solid fuels and cooking-only use, but tests for subgroup differences were not statistically significant (*p* > 0.05).

**Conclusion:**

After rigorous adjustment for publication bias, the available published evidence does not support the conclusion that indoor fuel use is an independent risk factor for atopic dermatitis. The weak association observed in the literature appears to be a statistical artifact, potentially influenced by systemic publication bias.

**Systematic review registration:**

https://www.crd.york.ac.uk/PROSPERO/view/CRD420251083253, identifier (CRD420251083253).

## 1 Introduction

Household air pollution (HAP) remains a formidable global public health challenge, particularly in low- and middle-income countries. The World Health Organization (WHO) estimates that approximately 2.1 billion people worldwide continue to rely on the combustion of solid fuels (e.g., wood, crop waste, charcoal, coal, and animal dung) and kerosene in open fires or inefficient stoves for daily cooking and heating (1). These combustion activities release a complex mixture of hazardous pollutants, including particulate matter (PM), sulfur dioxide (SO2), nitrogen oxides (NOx), and polycyclic aromatic hydrocarbons (PAHs). In poorly ventilated homes, concentrations of fine particulate matter (PM2.5) can reach levels up to 100 times higher than WHO-recommended limits (1). This pervasive exposure is estimated to cause 3.2 million premature deaths annually from noncommunicable diseases such as stroke, heart disease, chronic obstructive pulmonary disease (COPD), and lung cancer (1). Women and young children bear a disproportionate burden of this risk, as they typically spend the most time in the domestic environment near the sources of combustion.

Concurrently, atopic dermatitis (AD), also known as atopic eczema, has emerged as a significant and growing public health issue. AD is a common chronic inflammatory skin disease characterized by intense pruritus, a relapsing–remitting course, and a profound negative impact on patient quality of life and economic productivity (2). Data from the Global Burden of Disease (GBD) 2021 study indicated a rising global prevalence, with an estimated 129 million individuals affected by AD in 2021, representing a substantial increase in the absolute number of cases since 1990 (2). This trend underscores the urgent need to identify modifiable environmental risk factors that may contribute to its development and exacerbation.

A strong biological rationale supports a potential link between exposure to air pollutants from indoor fuel combustion and the pathogenesis of AD. A growing body of mechanistic evidence demonstrates that key pollutants found in HAP, such as PM and PAHs, can trigger or worsen AD through multiple pathways (3). These include disrupting the integrity of the epidermal barrier, inducing oxidative stress, and promoting cutaneous inflammation. Many of these effects are mediated by the activation of cellular signaling pathways, including the aryl hydrocarbon receptor (AhR), which is a key sensor of environmental toxins. These molecular events are central to the pathophysiology of AD, suggesting that exposure to indoor fuel emissions could plausibly increase disease risk (3).

Despite this biological plausibility, the body of epidemiological research examining the association between indoor fuel use and AD has yielded inconsistent and inconclusive results (4, 5). This inconsistency may stem from a range of methodological limitations in the primary studies, including variations in exposure assessment, control for confounding factors, and outcome definition (6, 7). Most importantly, no prior systematic review has quantitatively evaluated the potential impact of publication bias on the pooled estimate, a critical methodological concern in a field characterized by numerous observational studies with varying sample sizes. This evidence gap precludes a definitive conclusion on this topic and highlights the need for a more robust synthesis of the evidence.

To address this evidence gap, this study was designed to systematically review the available literature and conduct a meta-analysis to quantitatively synthesize the association between indoor fuel use and the risk of AD. A primary and distinguishing objective of this work was to move beyond a simple summary of the evidence by rigorously investigating the potential influence of publication bias and other methodological limitations within the primary studies. By critically appraising how these factors may have shaped the current evidence landscape, this study aims to provide a more nuanced and reliable conclusion on the topic.

## 2 Materials and methods

This systematic review and meta-analysis was conducted and reported following the guidelines of the Preferred Reporting Items for Systematic Reviews and Meta-Analyses (PRISMA) 2020 statement (8). To ensure transparency and minimize bias, the study protocol was prospectively registered in the International Prospective Register of Systematic Reviews (PROSPERO) under the registration number CRD420251083253.

### 2.1 Inclusion and exclusion criteria

Studies were included based on the following predefined criteria:

(1) Population: Human participants. To ensure the comprehensiveness and unbiased nature of this systematic review, our search strategy and inclusion criteria were designed without age restrictions. Although the final included studies are predominantly focused on children and adolescents, one study (5) enrolled participants aged 18–25, confirming that our target population was the general human population.
(2) Exposure: Indoor fuel combustion activities for cooking or heating, including the use of fuels such as coal, wood, charcoal, crop waste, animal dung, kerosene, ethanol, biogas, or natural gas.
(3) Comparison: A reference group with lower exposure, such as households using cleaner fuels (e.g., electricity) or technologies.
(4) Outcome: A diagnosis of atopic dermatitis or atopic eczema.
(5) Study Design: Original observational studies, including cohort and cross-sectional designs, that reported an effect estimate such as an odds ratio (OR), with a corresponding 95% confidence interval (CI), or provided sufficient raw data for their calculation.
(6) Language: Articles published in English.

Studies were excluded if they focused primarily on outdoor fuel combustion, environmental tobacco smoke (ETS) as the main exposure, or were non-original research (e.g., reviews, conference abstracts, letters to the editor). Animal studies and studies with insufficient data for extraction were also excluded.

### 2.2 Search strategy and literature screening

A comprehensive literature search was conducted in PubMed, Embase, Web of Science, and the Cochrane Library from their inception to May 28, 2025. The search strategy combined Medical Subject Headings (MeSH) and free-text terms related to both the exposure (e.g., “stove,” “cookstove,” “biomass,” “fuels,” “cooking,” “heating,” “coal,” “wood”) and the outcome (e.g., “Atopic Dermatitis,” “Atopic Eczema”). The detailed search strategy for each database is provided in Supplementary Table S1. The reference lists of included articles were also manually screened to identify additional relevant studies.

Two reviewers independently screened titles and abstracts to remove irrelevant articles, followed by a full-text review of potentially eligible studies to determine final inclusion. Any disagreements were resolved through discussion or consultation with a third reviewer.

### 2.3 Data extraction and quality assessment

Data were extracted independently by the same two reviewers using a standardized Excel spreadsheet. Extracted information included: first author, publication year, country, study design, sample size, participant characteristics (age), exposure type (fuel and usage), outcome definition and ascertainment method, and the most fully adjusted effect estimate with its 95% CI. When a single study reported multiple effect sizes for distinct, non-overlapping exposure subgroups (e.g., solid fuels vs. gas fuels, or cooking vs. heating) compared to a common reference group, each was treated as an independent effect size in our analysis. This approach allows for a more granular examination of different exposure scenarios. Consequently, 21 independent effect sizes were extracted from the 10 included studies.

The methodological quality of included studies was assessed independently by two reviewers. The Joanna Briggs Institute (JBI) Critical Appraisal Checklist for Analytical Cross-Sectional Studies was used for cross-sectional studies. This tool assesses eight domains, and studies with a score of ≥50% were considered for inclusion. For cohort studies, the Newcastle-Ottawa Scale (NOS) was used, which evaluates studies on three domains (selection, comparability, and outcome) with a maximum score of nine stars. Studies scoring seven or more stars were considered high quality. Detailed results of the quality assessment for each included study against each criterion are presented in Supplementary Table S2.

### 2.4 Statistical analysis

All statistical analyses were performed using the ‘meta’ package in R software (version 4.3.1). Odds ratios (ORs) and their 95% CIs were used as the primary effect measure as this was the metric reported by all included studies. It is important to note that while our analysis included one cohort study (9), it used multiple logistic regression analysis to control for confounders and thus also reported its findings as ORs. Therefore, no conversion or harmonization of different effect measures (e.g., from Relative Risk) was required. Statistical heterogeneity among studies was assessed using Cochran’s Q test (with a significance level of *p* < 0.10) and the I^2^ statistic, where I2 > 50% was interpreted as substantial heterogeneity. A random-effects model (Der Simonian-Laird method) was used for the primary meta-analysis to account for anticipated between-study variance (10).

#### 2.4.1 Assessment of publication bias

A comprehensive assessment of publication bias was conducted. This included visual inspection of the funnel plot for asymmetry and a suite of formal statistical tests: Begg’s rank correlation test, Egger’s linear regression test, Peters’ test, Deeks’ test, Macaskill’s test, and Tang’s test (11, 12).

#### 2.4.2 Bias correction and sensitivity analyses

If evidence of bias was detected, two distinct correction methods were employed. First, the non-parametric Duval and Tweedie’s trim-and-fill method was used to estimate the number of potentially missing studies and provide an adjusted pooled effect estimate. Second, as a more sophisticated approach, parametric selection models were fitted to adjust the pooled estimate based on the probability of study publication, which is often related to the study’s *p*-value (13). To assess the robustness of the findings, a leave-one-out sensitivity analysis was performed by sequentially removing one study at a time and recalculating the pooled OR.

#### 2.4.3 Subgroup analyses

Finally, subgroup analyses were conducted based on fuel type (solid vs. gas) and fuel usage (cooking vs. heating) to investigate potential sources of heterogeneity. Statistical significance was set at *p* < 0.05 for all analyses except for the test of heterogeneity (14).

## 3 Results

### 3.1 Literature search results

**Figure 1:**
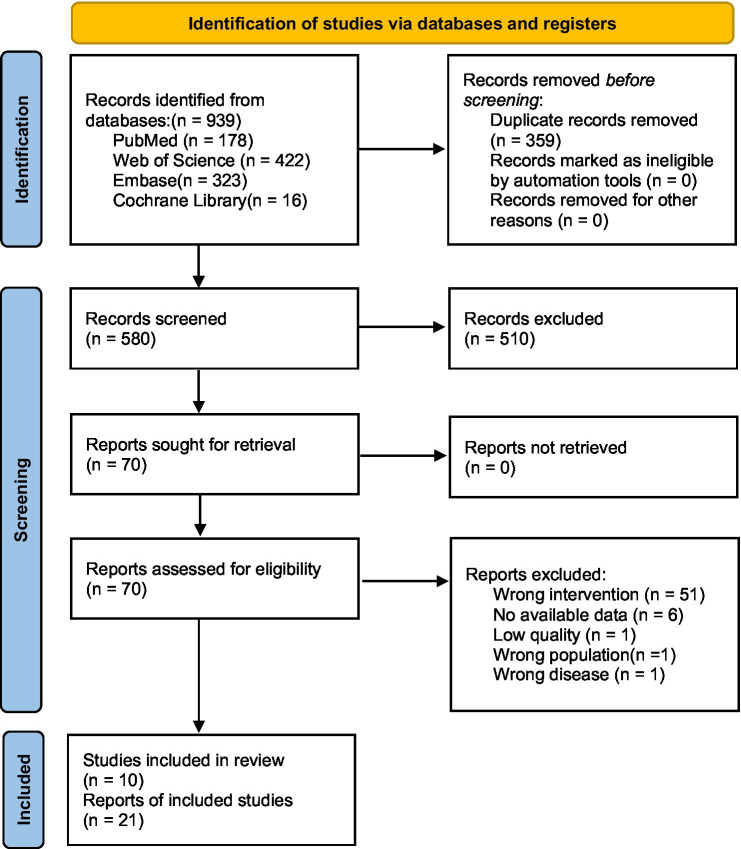
PRISMA flow diagram of study selection.

The initial database search yielded 939 articles. After removing 359 duplicates, 580 unique records remained. A review of titles and abstracts led to the exclusion of 510 articles that were clearly not relevant. The full texts of the remaining 70 articles were assessed for eligibility, of which 60 were excluded for reasons such as having an irrelevant exposure or outcome, being a review article, or lacking sufficient data. Ultimately, 10 studies met the inclusion criteria and were included in the meta-analysis. The PRISMA flow diagram detailing the study selection process is shown in Figure 1.

### 3.2 Basic characteristics of included studies

The 10 included observational studies were published between 1996 and 2024 and comprised a total sample size of 53,924 participants. The studies included nine cross-sectional designs and one prospective cohort study. They were geographically diverse, conducted in South Africa, China, Japan, Spain, Ukraine, Australia, Finland, and Germany. Most studies used the International Study of Asthma and Allergies in Childhood (ISAAC) questionnaire for outcome ascertainment, and all studies adjusted for basic confounders such as age and sex. The quality assessment indicated that the overall methodological quality of the included studies was acceptable; all cross-sectional studies had JBI scores above 50%, and the single cohort study was rated as high quality with a NOS score of 8 out of 9 stars. The detailed characteristics and quality assessments are presented in Tables 1, 2, respectively.

**Table 1:** Quality assessment of included studies.

Author	Study type	Tool	Score/Rating
Mandla Bhuda et al. (4)	Cross-sectional	JBI	8/8 (100%)
Zhang, R. et al. (22)	Cross-sectional	JBI	8/8 (100%)
Ukawa, S. et al. (23)	Cross-sectional	JBI	8/8 (100%)
Vicedo-Cabrera, A. M. et al. (18)	Cross-sectional	JBI	8/8 (100%)
Fedortsiv, O. et al. (19)	Cross-sectional	JBI	8/8 (100%)
Zhang, G. et al. (G. (17))	Cross-sectional	JBI	8/8 (100%)
Kilpeläinen, M. et al. (5)	Cross-sectional	JBI	7/8 (87.5%)
Schäfer, T. et al. (24)	Cross-sectional	JBI	8/8 (100%)
Schäfer, T. and Vieluf, D. (20)	Cross-sectional	JBI	8/8 (100%)
Miyake, Y. et al. (9)	Cohort	NOS	8/9 (High Quality)

**Table 2:** Characteristics of included studies.

Author	Publication year	Country	Study type	Sample size	Age	Exposure type	Outcome ascertainment	OR (95% CI)
Mandla Bhuda et al.	2024	South Africa	Cross-sectional	1840	6–7	Gas	ISAAC Questionnaire	1.63 (1.00, 2.65)
Mandla Bhuda et al.	2024	South Africa	Cross-sectional	1840	6–7	Open flame (paraffin/wood/coal)	ISAAC Questionnaire	1.94 (1.00, 3.74)
Zhang, R. et al.	2023	China	Cross-sectional	5,730	3–6	Coal	ISAAC Questionnaire	1.28 (1.03, 1.60)
Zhang, R. et al.	2023	China	Cross-sectional	5,730	3–6	Wood	Qualitative questionnaire	1.40 (1.01, 1.95)
Zhang, R. et al.	2023	China	Cross-sectional	5,730	3–6	Gas	Qualitative questionnaire	0.91 (0.73, 1.14)
Ukawa, S. et al.	2013	Japan	Cross-sectional	4,254	6–12	Non-electric heating (vented)	ISAAC Questionnaire	1.28 (0.93, 1.80)
Ukawa, S. et al.	2013	Japan	Cross-sectional	4,254	6–12	Non-electric heating (unvented)	ISAAC Questionnaire	1.45 (1.01, 2.11)
Vicedo-Cabrera, A. M. et al.	2012	Spain	Cross-sectional	21,355	6–7	Gas	ISAAC Questionnaire	1.10 (0.95, 1.28)
Vicedo-Cabrera, A. M. et al.	2012	Spain	Cross-sectional	21,355	6–7	Biomass furnace	ISAAC Questionnaire	1.51 (0.60, 3.18)
Vicedo-Cabrera, A. M. et al.	2012	Spain	Cross-sectional	21,355	6–7	Biomass heating	ISAAC Questionnaire	1.02 (0.82, 1.25)
Vicedo-Cabrera, A. M. et al.	2012	Spain	Cross-sectional	21,355	6–7	Biomass furnace or heating	ISAAC Questionnaire	1.00 (0.82, 1.25)
Vicedo-Cabrera, A. M. et al.	2012	Spain	Cross-sectional	21,355	6–7	Gas stove or kerosene heating	ISAAC Questionnaire	1.01 (0.86, 1.19)
Vicedo-Cabrera, A. M. et al.	2012	Spain	Cross-sectional	21,355	6–7	Gas/Kerosene/Paraffin heating	ISAAC Questionnaire	0.91 (0.80, 1.04)
Fedortsiv, O. et al. ^1^	2012	Ukraine	Cross-sectional	4,871	6–14	Wood or coal	Physician-diagnosed	1.20 (0.63, 2.28)
Miyake, Y. et al.	2007	Japan	Cohort	865	2–9 months	Gas	Qualitative questionnaire	1.67 (0.48, 10.64)
Miyake, Y. et al.	2007	Japan	Cohort	865	2–9 months	Unspecified heating fuel	Qualitative questionnaire	1.65 (0.92, 3.15)
Zhang, G. et al.	2006	Australia	Cross-sectional	977	4–12	Gas	ISAAC Questionnaire	1.74 (1.05, 2.86)
Kilpeläinen, M. et al.	2001	Finland	Cross-sectional	8,480	18–25	Wood	Self-reported physician diagnosis	1.02 (0.82, 1.28)
Schäfer, T. et al.	1999	Germany	Cross-sectional	1842	5–14	Wood	Dermatological exam	1.22 (0.50, 2.90)
Schäfer, T. et al.	1999	Germany	Cross-sectional	1842	5–14	Gas	Dermatological exam	1.15 (0.21, 4.04)
Schäfer, T. et al.	1996	Germany	Cross-sectional	1,273	5–7	Gas	Dermatological exam	1.68 (1.11, 2.56)

### 3.3 Meta-analysis of indoor fuel use and atopic dermatitis risk

**Figure 2:**
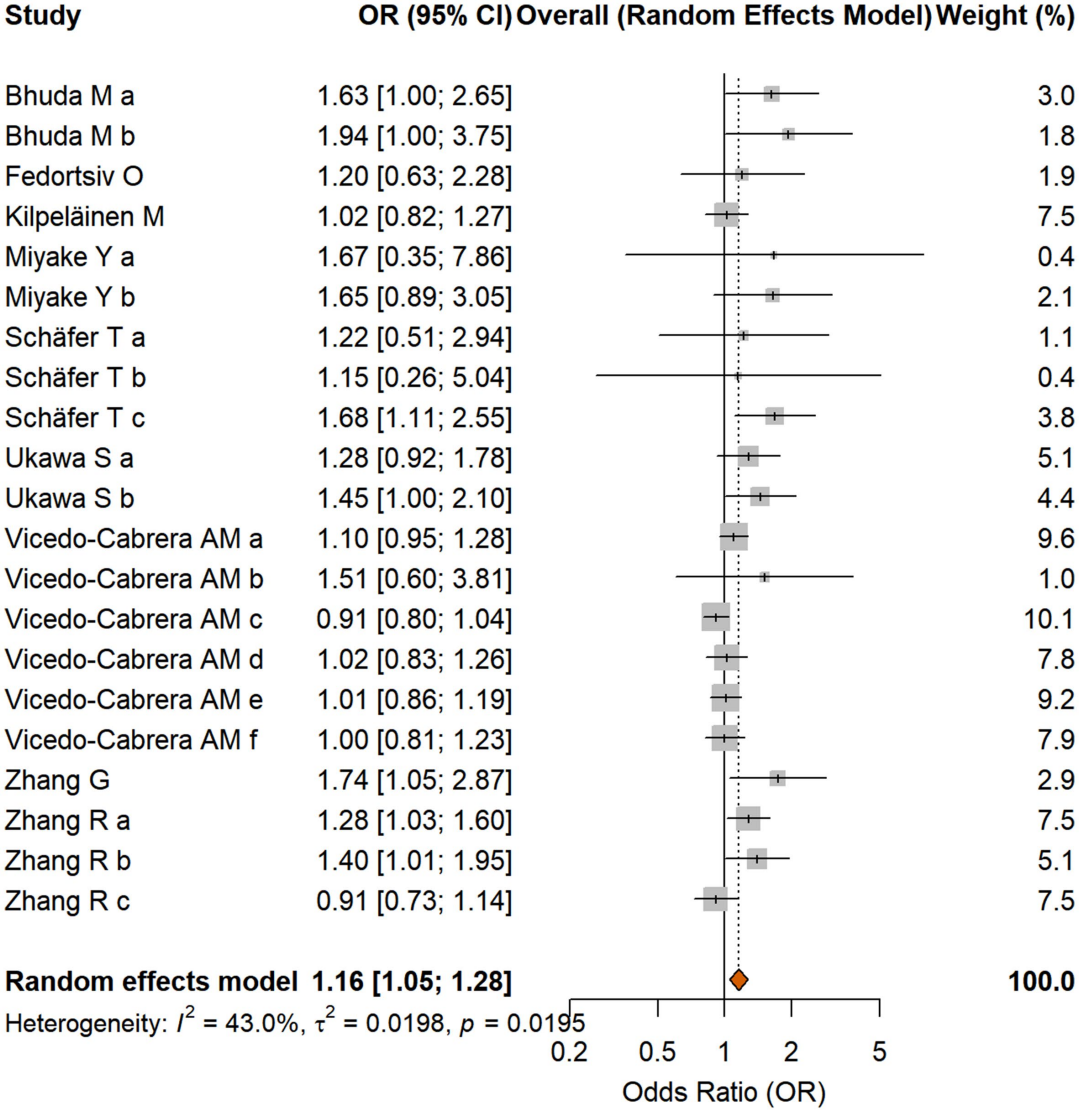
Forest plot of the association between indoor fuel use and atopic dermatitis. The letter suffixes for studies that contributed multiple effect sizes represent the following specific exposures: Bhuda M: a = Gas; b = Open flame (paraffin/wood/coal). Miyake Y: a = Gas; b = Unspecified heating fuel. Schäfer T: a = Wood (1999); b = Gas (1999); c = Gas (1996). Ukawa S: a = Non-electric heating (vented); b = Non-electric heating (unvented). Vicedo-Cabrera AM: a = Gas(cook); b = Biomass furnace(cook); c = Gas/Kerosene/Paraffin(heating); d = Biomass(heating); e = Gas/Kerosene/Paraffin(cook and heating); f = Biomass(cook and heating). Zhang R: a = Coal; b = Wood; c = Gas.

The random-effects meta-analysis, combining 21 effect sizes from the 10 studies, indicated a statistically significant positive association between indoor fuel use and the risk of AD. Several publications contributed more than one effect size to the analysis, as they assessed different types of indoor fuel exposures (e.g., solid fuels vs. gas) or different usage patterns (e.g., cooking vs. heating) separately. The forest plot in Figure 2 presents all 21 independent effect sizes extracted from the 10 included studies. The pooled OR was 1.158 (95% CI [1.051, 1.276], *p* = 0.0031). The analysis revealed moderate heterogeneity among the studies (*I*^2^ = 43.0%, Cochran’s Q test *p* = 0.0195), supporting the decision to use a random-effects model. The forest plot summarizing the individual study results and the pooled estimate is shown in Figure 2.

### 3.4 Publication bias assessment

**Figure 3:**
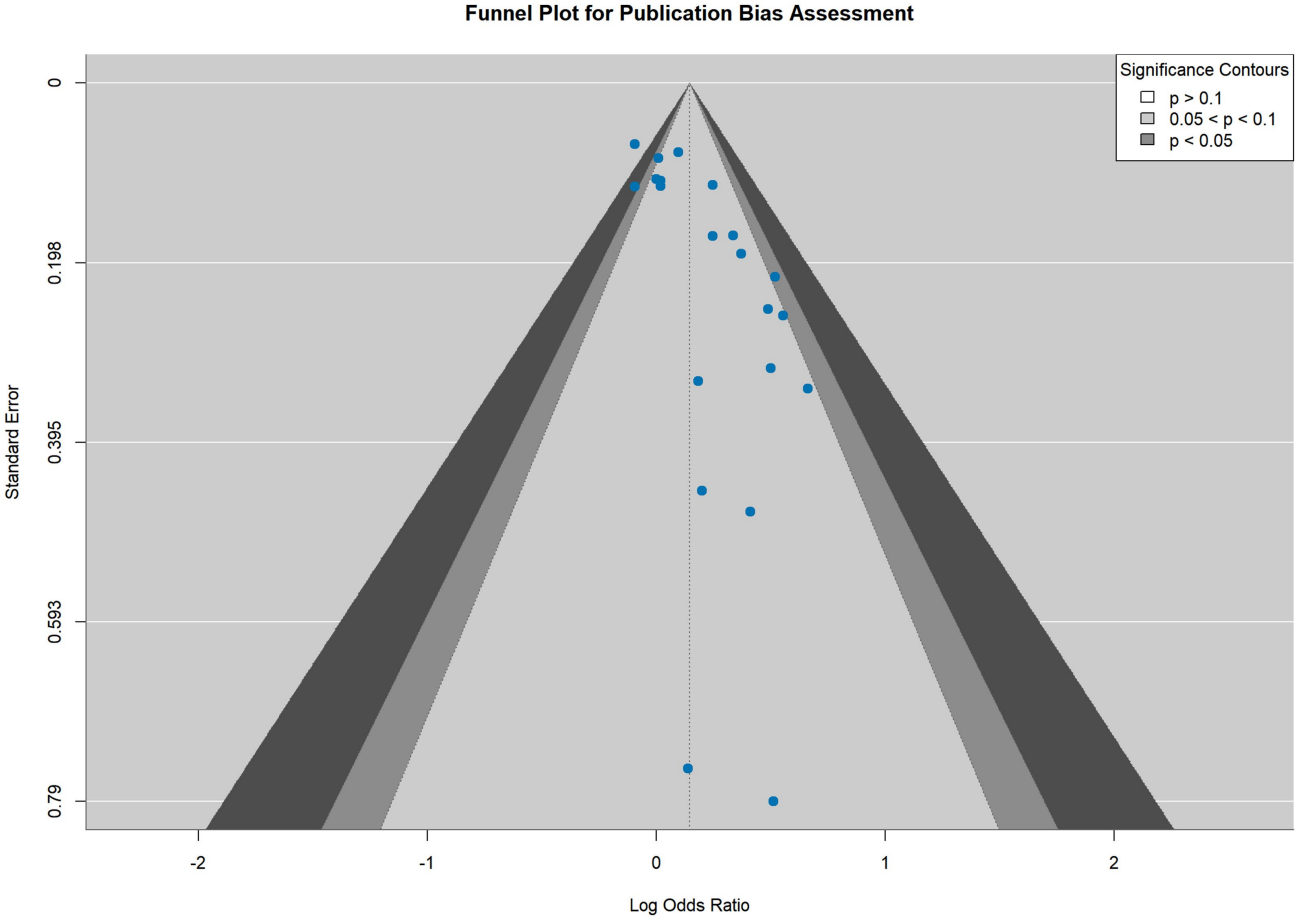
Funnel plot of included studies.

Visual inspection of the funnel plot (Figure 3) revealed asymmetry, with a noticeable lack of studies in the bottom-left portion of the plot. This pattern suggests an absence of small studies reporting null or negative associations, a classic indicator of “small-study effects,” which may arise from publication bias.

This visual finding was corroborated by a comprehensive panel of statistical tests (Table 3). While Begg’s rank correlation test was borderline (*p* = 0.0533), Egger’s linear regression test showed highly significant asymmetry (*p* = 0.0006). This conclusion was strongly supported by Peters’ test (*p* < 0.0001), Deeks’ test (*p* < 0.0001), Macaskill’s test (*p* = 0.0006), and Tang’s test (*p* = 0.0006). The strong convergence of results from multiple tests, which rely on different statistical assumptions, provides robust evidence that the included literature likely represents a biased sample of all studies conducted on this topic.

**Table 3:** Statistical tests for publication bias.

Method	*p*-value	Conclusion
Begg’s rank correlation test	0.0533	Asymmetry not statistically significant
Egger’s regression test	0.0006	Significant asymmetry detected
Peters’ test	< 0.0001	Significant asymmetry detected
Macaskill’s test	0.0006	Significant asymmetry detected
Deeks’ test	< 0.0001	Significant asymmetry detected
Tang’s test	0.0006	Significant asymmetry detected

### 3.5 Bias correction and sensitivity analyses

To quantify the impact of the detected publication bias, two different correction methods were applied. The results, summarized in Table 4, demonstrate that the initial significant finding was not robust to bias adjustment.

**Table 4:** Comparison of original and bias-corrected pooled effect estimates.

Method	Adjusted OR	Adjusted 95% CI
Original random-effects model	1.158	1.051–1.276
Trim and fill	1.040	0.930–1.164
Standard selection model	1.000	0.992–1.008
Flexible selection model	1.000	0.993–1.007
Multi-level selection model	1.085	0.991–1.188

**Figure 4:**
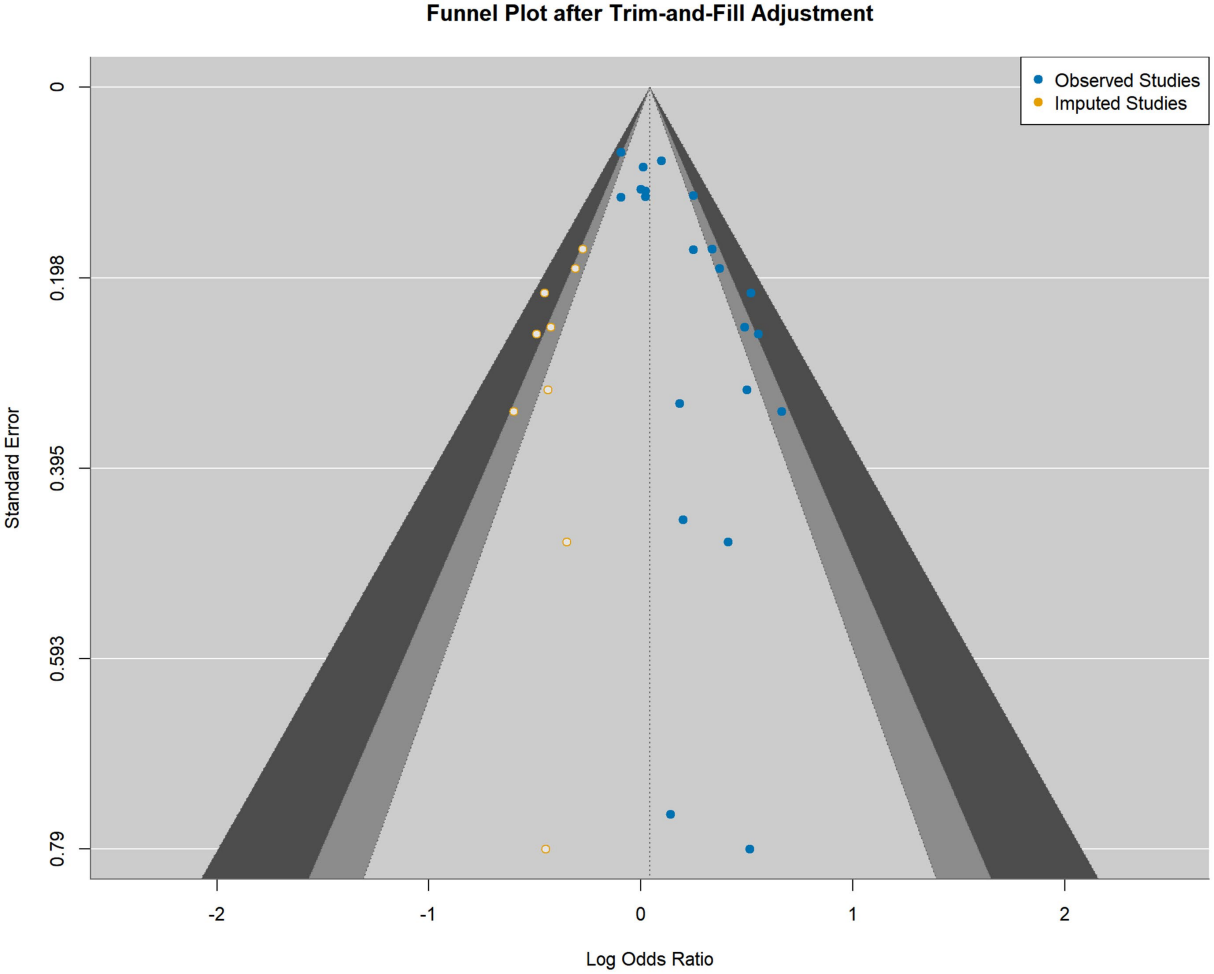
Funnel plot after trim-and-fill adjustment.

First, the non-parametric trim-and-fill method estimated that nine studies were missing from the analysis due to bias. After imputing these hypothetical studies to create a more symmetric funnel plot (Figure 4), the adjusted pooled OR was reduced to 1.040 (95% CI [0.930, 1.164]), which is no longer statistically significant.

Second, parametric selection models, which explicitly model the probability of study publication, were employed. These models consistently attenuated the effect estimate to the null. The standard single-tailed selection model, for instance, yielded an adjusted OR of 1.000 (95% CI [0.992, 1.008]). The convergence of these methodologically distinct approaches provides strong evidence that the initial association was likely a statistical artifact driven by selective publication.

**Figure 5:**
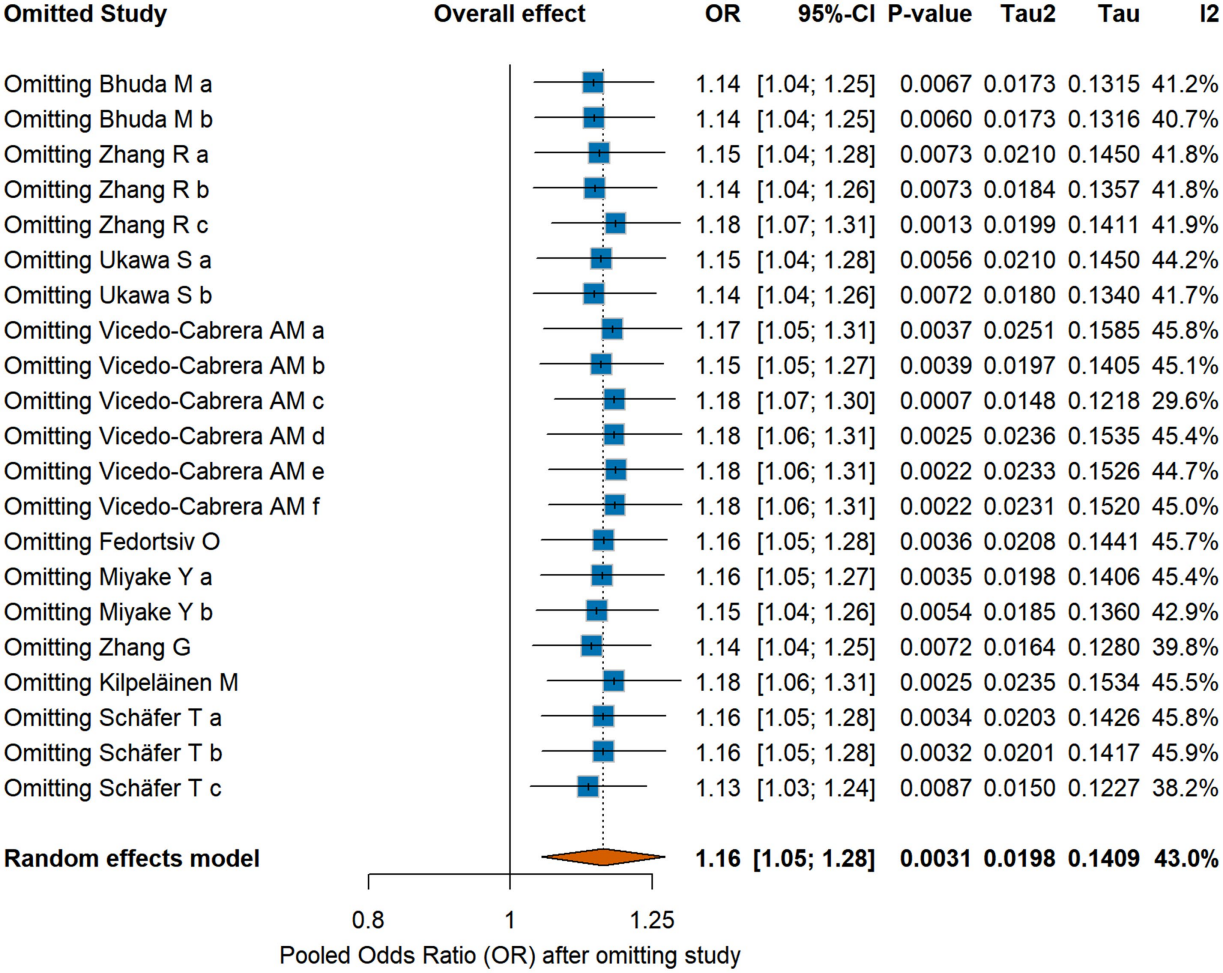
Leave-one-out sensitivity analysis.

In contrast, the leave-one-out sensitivity analysis (Figure 5) showed that the initial uncorrected result was stable. Sequentially removing each study did not substantially alter the pooled OR (range: 1.13 to 1.18) or its statistical significance, indicating that the unadjusted finding was not unduly influenced by any single study.

### 3.6 Subgroup analyses

**Figure 6:**
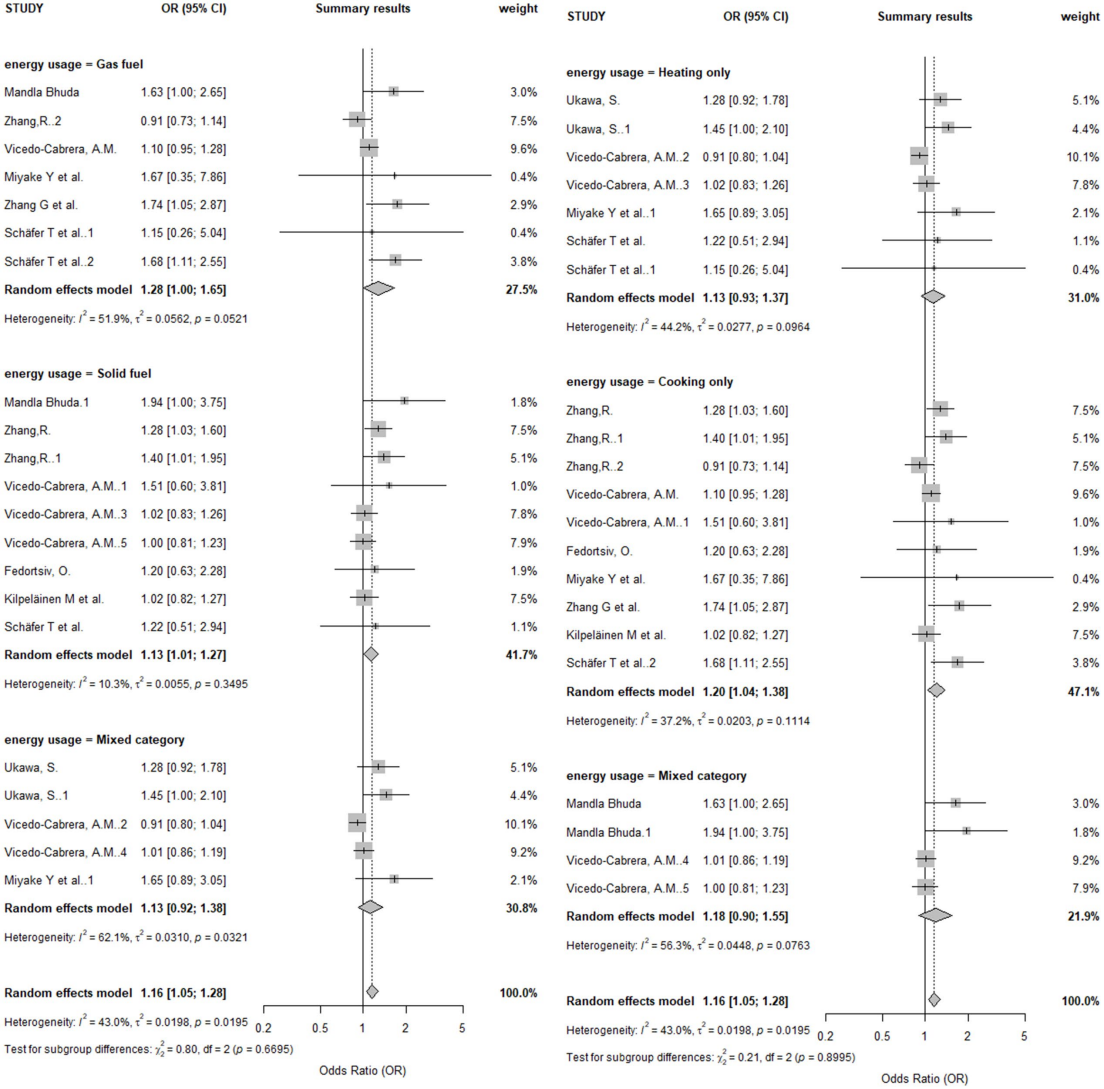
Forest plot of subgroup analysis.

Subgroup analyses were conducted to explore potential sources of heterogeneity (Table 5). The results are presented in a forest plot in Figure 6. When stratified by fuel type, a significant association was observed for solid fuels (OR = 1.13, 95% CI [1.01, 1.27]), but not for gas fuels (OR = 1.28, 95% CI [1.00, 1.65]). However, the test for a difference between these subgroups was not statistically significant (*p* = 0.670), indicating no clear evidence that the risk differs by fuel type.

**Table 5:** Subgroup analysis results.

Subgroup classification	No. of effect sizes (k)	Pooled OR (95% CI)	Subgroup *p*-value	I^2^ (%)
Energy type				
Gas fuel	7	1.28 [1.00, 1.65]	0.054	51.9%
Solid fuel	9	1.13 [1.01, 1.27]	0.034	10.3%
Both	5	1.22 [0.92, 1.38]	0.247	62.1%
Test for subgroup differences			p = 0.670	
Use type				
Heating only	7	1.13 [0.93, 1.37]	0.205	44.2%
Cooking only	10	1.20 [1.04, 1.38]	0.013	37.2%
Both	4	1.18 [0.90, 1.55]	0.218	56.3%
Test for subgroup differences			p = 0.900	
Overall	21	1.16 [1.05, 1.28]	0.001	42.8%

When stratified by fuel usage, a significant association was found for cooking only (OR = 1.20, 95% CI [1.04, 1.38]), while no significant association was found for heating only or for combined heating and cooking. Similarly, the test for subgroup differences was not statistically significant (*p* = 0.900).

## 4 Discussion

### 4.1 Principal finding: an association undermined by publication bias

This systematic review and meta-analysis provides the first quantitative synthesis of the association between indoor fuel use and the risk of atopic dermatitis. The initial analysis of published literature suggested a modest, statistically significant 16% increase in the odds of AD associated with exposure to indoor fuel combustion (OR = 1.158). However, this finding proved to be fragile. A comprehensive and multi-pronged assessment revealed compelling evidence of publication bias, suggesting that studies with smaller or null effects were less likely to have been published. When this bias was statistically corrected using both non-parametric (trim-and-fill) and parametric (selection models) methods, the pooled effect estimate was attenuated to the null, and its statistical significance was completely eliminated. Therefore, the central conclusion of this study is that the weak positive association observed in the published literature is likely a statistical artifact, heavily influenced by the selective reporting of positive findings. Based on a rigorous, bias-adjusted analysis of the currently available evidence, it is not possible to conclude that indoor fuel use is an independent risk factor for AD.

### 4.2 The potential role of exposure misclassification

The subgroup analyses yielded a counterintuitive result: no statistically significant difference in risk was detected between solid fuels and gas fuels, despite the well-established fact that solid fuel combustion releases far higher levels of harmful pollutants, particularly PM2.5. This finding does not necessarily imply that the risks are equivalent (15). Instead, it may reflect a profound methodological limitation common to the included primary studies: potential exposure misclassification (16).

While this bias is a concern in any observational design, it is especially pertinent here because the vast majority of studies that form our evidence base relied on questionnaires and long-term recall for exposure assessment. This retrospective assessment is highly susceptible to recall error, leading to non-differential exposure misclassification (15). A well-documented statistical consequence of such misclassification is a bias of the effect estimate (e.g., the OR) toward the null value of 1.0, potentially masking a true effect.

This reliance on recall is not a hypothetical concern but a consistent feature across the literature. For instance, studies by Zhang et al. (17), Vicedo-Cabrera et al. (18), Fedortsiv et al. (19), and the recent work by Bhuda et al. (4) all collected key exposure data on cooking fuels and home environments via parental questionnaires. Even earlier studies, such as Schäfer et al. (20), did the same. The inherent imprecision of asking a parent to recall details from months or years ago is a key mechanism that could lead to an underestimation of the true effect. Therefore, this measurement error, rooted in the design of the primary studies, serves as a core alternative explanation for our null finding after correcting for publication bias.

This type of imprecise measurement can lead to non-differential exposure misclassification, meaning that exposed and unexposed individuals are misclassified at similar rates. The well-documented statistical consequence of such misclassification in epidemiological studies is a bias of the effect estimate (e.g., the OR) toward the null value of 1.0. While this concept is most clearly demonstrated in cohort studies, it can also be a significant issue in cross-sectional designs that rely on long-term recall of exposure, potentially masking a true effect (21).

### 4.3 Alternative explanations and unaddressed confounding

Beyond publication bias and exposure misclassification, several other factors could explain the null finding after bias correction. First, residual confounding is a major concern. Socioeconomic status, a strong determinant of both household fuel choice and health outcomes, may not have been adequately controlled for in all primary studies. Factors such as parental education, household income, and housing quality could confound the observed association, and the adjustments made in original studies might be insufficient. Second, co-exposure to other environmental pollutants was often not accounted for. Exposure to ambient air pollution or environmental tobacco smoke, which are often correlated with indoor fuel use, could independently affect AD risk and thus confound the results. Finally, potential misclassification of the outcome warrants consideration. The reliance on self-reported questionnaires like the ISAAC protocol, rather than a physician’s clinical diagnosis, in the majority of included studies could lead to non-differential misclassification of AD status, which would also tend to weaken any true association and bias the result toward the null.

### 4.4 Strengths and limitations of this meta-analysis

The primary strength of this study lies in its comprehensive and rigorous approach to assessing and correcting for publication bias. By employing a wide array of statistical tests and multiple, methodologically distinct adjustment techniques, this analysis provides a robust evaluation of the stability of the published evidence. Further strengths include a systematic and reproducible search strategy, adherence to PRISMA reporting guidelines, and prospective protocol registration.

However, this review is subject to several limitations, most of which are inherited from the primary studies. First, the predominance of cross-sectional designs prevents any inference of causality, as it is impossible to establish whether the exposure preceded the onset of AD. Second, although moderate, the presence of statistical heterogeneity suggests that there are underlying differences between the studies that were not fully explained by the subgroup analyses. Third, the review was restricted to English-language publications, which may have introduced language bias. Finally, and most importantly, our conclusions are drawn from a body of literature constrained by the potential methodological weaknesses discussed above, including exposure misclassification, residual confounding, and outcome measurement error.

### 4.5 Implications for future research

The findings of this meta-analysis highlight critical gaps and provide clear direction for future research in this area. To move the field forward and obtain a definitive answer, studies must overcome the methodological shortcomings of the past. The following recommendations are proposed:

1. Adopt Prospective Cohort Designs: Future studies should employ prospective, longitudinal designs that enroll participants before the onset of disease. This is essential for establishing the correct temporal sequence between exposure and outcome and for making stronger causal inferences.
2. Improve Exposure Assessment: There is an urgent need to move beyond simplistic, questionnaire-based exposure assessment. Future research should incorporate more objective and quantitative methods to reduce misclassification. This could include:
  Direct Measurement: Utilizing low-cost indoor air quality monitors to measure key pollutants like PM2.5, NO2, and CO within a subsample of homes to validate and calibrate exposure models.
  Advanced Modeling: Employing sophisticated exposure models, such as Land-Use Regression (LUR) or satellite-based models, to estimate the contribution of ambient pollution to the indoor environment, combined with detailed housing and ventilation data.
  Personal Monitoring: Using wearable personal exposure monitors in a subset of participants to capture true breathing-zone concentrations, which account for individual time-activity patterns and mobility.

By implementing these more rigorous designs, future research will be better positioned to accurately characterize the dose–response relationship and determine whether a true causal link exists between indoor fuel combustion and the risk of atopic dermatitis.

## 5 Conclusion

Based on a systematic review and a rigorous, multi-faceted analysis that corrected for substantial publication bias, the currently available published evidence does not support the conclusion that indoor fuel use is an independent risk factor for atopic dermatitis. The weak positive association observed in the unadjusted meta-analysis is likely a statistical artifact driven by the preferential publication of studies with positive results. Furthermore, the primary literature is hampered by significant methodological limitations, including potential exposure misclassification, uncontrolled confounding, and imprecise outcome assessment, which prevents a reliable assessment of any potential dose–response relationship. Future research must employ more robust methodologies, particularly prospective cohort designs with quantitative and validated exposure assessment, to definitively clarify the relationship between household air pollution and this common and burdensome disease.

## Data Availability

The original contributions presented in the study are included in the article/Supplementary material, further inquiries can be directed to the corresponding author.
